# Glucose metabolism disorders and changes in cardiovascular risk among adult Peruvian population

**DOI:** 10.12688/wellcomeopenres.22701.2

**Published:** 2025-11-12

**Authors:** Jhohann Sedano-Espinoza, Kevin Perez-Ochoa, Erick Yalico-Quispe, Antonio Bernabe-Ortiz

**Affiliations:** 1 Universidad Cientifica del Sur, Miraflores, Peru

**Keywords:** Type 2 diabetes mellitus, prediabetes, cardiovascular risk, blood pressure

## Abstract

**Background:**

Rates of cardiovascular diseases are increasing annually, and glucose metabolism disorders play an important role in cardiovascular risk. This study aimed to assess changes in cardiovascular risk over time according to the levels of blood glucose, especially prediabetes and type 2 diabetes.

**Methods:**

Prospective cohort study conducted in the northern Peru. Two were the outcomes of interest, evaluated at the cohort’s baseline and six years after: cardiovascular risk (in percentage), defined using the laboratory-free risk score (HEARTS from the World Health Organization), and the mean arterial pressure (in mmHg); whereas the exposure was glucose metabolism disorder, especially prediabetes and type 2 diabetes, compared to normoglycaemia. Associations were evaluated using mixed linear regression models, and coefficients (β) and 95% confidence intervals (95% CI) were reported.

**Results:**

A total of 1607 subjects were recruited, with a mean age of 48.0 (standard deviation [SD]: 10.5) years, and 50.3% women. Baseline prevalence of prediabetes and type 2 diabetes was 16.9% (95%CI: 15.1%-18.9%) and 11.0% (95%CI: 9.5%-12.6%), respectively; whereas the average of the cardiovascular risk and mean arterial pressure was 3.0% (SD: 2.5%) and 93.1 (SD: 11.9) mmHg, respectively. At baseline, those with prediabetes and type 2 diabetes had a higher cardiovascular risk (0.31% and 0.33%, respectively) and a higher mean arterial pressure (3.3 and 4.2 mmHg, respectively) than those with normal glycaemia. No significant change was found in cardiovascular risk between baseline and follow-up; however, there was a reduction in mean arterial pressure levels, greater among those with normoglycaemia than in cases of prediabetes and type 2 diabetes.

**Conclusions:**

Glucose metabolism disorders were associated with an increased cardiovascular risk and mean arterial pressure at baseline, but only with higher levels of mean arterial pressure at follow-up. These findings suggest the need for cardiovascular risk assessment in patients with prediabetes and type 2 diabetes.

## Plain Language Summary

Subjects with alteration of the glucose metabolism, especially those with prediabetes and diabetes are at high cardiovascular risk. This is evaluated in this manuscript using two different indicators, a cardiovascular risk score (estimated using the WHO HEARTS tool) and the mean arterial pressure (estimated using the measurements of blood pressure). At the baseline of the study, individuals with prediabetes and diabetes had high cardiovascular risk and mean arterial pressure compared to those with normal glycaemia. At follow-up, on average, 6 years later, no change in cardiovascular risk and a reduction of mean arterial pressure was reported. Mean arterial pressure was higher among those with prediabetes and diabetes.

## Introduction

The number of subjects with cardiovascular diseases (i.e., ischemic heart disease, stroke and others related conditions) is increasing every year, causing 17.3 million deaths annually worldwide, and being considered one of the leading causes of mortality ^1^. According to the World Health Organization (WHO), cardiovascular diseases are the leading cause of death in middle-income countries ^2^, such as Peru.

The increase in cardiovascular events is secondary to the presence of risk factors, which can be classified as modifiable and non-modifiable ^3^. Latin America, including Peru, is no exception since its population presents a considerable increase in these risk factors ^4^. For example, a population-based study using data from the National Demographic and Family Health Survey (ENDES in Spanish) reported that the predicted 10-year absolute cardiovascular risk was, on average, 4.5% and 7.8% of the general population had a cardiovascular risk over 10% ^5^. In addition, the prevalence of hypertension, obesity and smoking, recognized cardiovascular risk factors, has been estimated to be 22.1%, 25.6% and 16.2%, respectively, in 2022 ^6^.

Simultaneously, glucose metabolism disorders have also increased in recent years globally ^7^, and Peru is on the same line ^8^. Thus, the national prevalence of type 2 diabetes was estimated at 7.0% in 2012, and in Lima it reached 8.4% ^9^, whereas the prevalence of prediabetes fluctuates between 18% and 22% ^9, 10^. However, in the north of the country, specifically in Tumbes, the prevalence of type 2 diabetes was 11.0% ^9^, whereas the prevalence of prediabetes may reach levels of 24.0% ^11^. The disorders of glucose metabolism cause a subsequent insulin resistance with cardiovascular involvement ^12, 13^. The WHO HEARTS technical package offers a strategic approach to assess and improve cardiovascular health in different contexts and consists of six modules and an implementation guideline, which can be useful for assessing how people's cardiovascular risk varies at the population level but also over time.

Because the prevalence of prediabetes and type 2 diabetes continues to increase in our context, it is necessary to determine the impact of impaired glucose metabolism on cardiovascular risk at the population level, especially in countries with limited economic resources and infrastructure, such as Peru. The most current Peruvian guidelines for the management of type 2 diabetes focuses on its diagnosis and risk factors at the primary care system level ^14^, but do not promote the management of subjects with elevated cardiovascular risk, especially those with prediabetes.

Therefore, the aim of the present study was to evaluate the change of cardiovascular risk, evaluated by a risk score and mean arterial pressure, according to the levels of glucose metabolism disorder, especially prediabetes and type 2 diabetes.

## Methods

### Study design

This is a prospective longitudinal cohort study. The baseline was conducted between 2016–2017 and aimed to evaluate the diagnostic accuracy of the Finnish Diabetes Risk Score (FINDRISC) and compare its performance with other risk scores ^15^. A new evaluation of participants was conducted during 2023, about six years later, by re-contacting all participants who were originally recruited at the baseline.

### Study setting and participants

The baseline study was conducted in a representative sample of a peri-urban area of Tumbes, a region located in northern Peru. This setting was chosen because the prevalence of obesity, according to BMI (32% vs. 18%) and type 2 diabetes, according to fasting plasma glucose (10% vs. 7%), was above the national average ^11^.

The baseline of the study enrolled adult individuals of both sexes, aged between 30 and 69 years, full-time residents (≥6 months) of the study area, eligible for procedures, and who consented participation. We excluded women who reported being pregnant at the moment of enrolment and subjects with some physical disability precluding body measurements (standing height, weight, or waist circumference).

For the present analysis, data from the original study were used provided they contained complete information on the variables of interest (glucose metabolism disorder, variables used for cardiovascular risk estimation and blood pressure measurements). For the prospective analysis, only subjects with complete data at follow-up were considered.

### Sampling

Participants were recruited using a single-stage random probability approach, stratified by sex. Data from the most current census at the moment of the cohort’s baseline (2014) were used for the sampling. Only one participant per household was enrolled in the study.

Power calculation was conducted using OpenEpi. OpenEpi is free and open-source software for epidemiologic statistics. It can be run from a web server or downloaded and run without a web connection ^16^. Assuming that the number of people with type 2 diabetes at baseline was 176 and the number of people with normoglycaemia was 1159, there was close to 100% power to find a difference in cardiovascular risk of 5%. In the case of prediabetes, as the number of cases was greater (n = 272), study power was also close to 100% to find a difference in cardiovascular risk of 5% ^10, 15^.

### Definition of variables

Two were our outcome variables related to cardiovascular risk. The first one was estimated using the risk score WHO HEARTS without laboratory tests, which assesses a person's 10-year cardiovascular risk and is calculated as a percentage using the variables age, sex, smoking status, systolic blood pressure and body mass index (BMI) values ^17, 18^. The cardiovascular risk was calculated for both baseline and follow-up, using the STATA command *whocvdrisk*. A free version of the risk calculator is available in Pan-American Health Organization (PAHO) webpage ^19^. The second outcome was the mean arterial pressure (MAP), calculated using values of systolic (SBP) and diastolic (DBP) blood pressure, estimated at both baseline and follow-up, using the following formula ^20^:

MAP=[SBP−DBP]/3+DBP

Glucose metabolism disorder was the exposure of interest, measured at the cohort’s baseline, and defined according to results of the oral glucose tolerance test ^21^, split into three groups: normoglycaemia (preprandial [fasting] glucose <100 mg/dl and postprandial glucose <140 mg/dl); prediabetes (preprandial glucose between 100 mg/dl and 125 mg/dl, or postprandial glucose between 140 mg/dl and 199 mg/dl); and type 2 diabetes (preprandial glucose ≥126 mg/dl or postprandial glucose ≥200 mg/dl or previous diagnosis of type 2 diabetes made by a physician).

Covariates used for analyses were: age (<40, 40-49, 50-59 or >60 years), sex (male or female), education level (>7, 7-11 and ≥12 years, representing primary, secondary and superior education, respectively), socioeconomic level, based on the household assets and divided into tertiles (low, medium or high), and health insurance (self-report of having or not to health insurance). Additionally, we also included behavioural variables such as smoking (self-reported consumption of at least 1 cigarette per day); alcohol consumption, self-reported and based on the number of times the participant consumed at least six bottles of beer (or equivalent) in the month prior to the survey (≤1 or >1 per month); level of physical activity, based on the International Physical Activity Questionnaire (IPAQ), and categorized according to the number of metabolic equivalents per minute in a week (low vs. moderate/high) ^22^; fruit and vegetable intake assessed by self-report of consumption of at least 1 fruit or vegetable per day (yes vs. no); presence of obesity, based on body mass index (<30 or ≥30 kg/m ^2^); and arterial hypertension (SBP ≥140 mmHg or SBP ≥90 mmHg or history of previous diagnosis of arterial hypertension) ^23^. These latter two variables were only used for descriptive purposes.

### Procedures

Participants provided written informed consent before commencing data collection. Data were collected using tablets, and trained personnel were responsible for the application of questionnaires (face-to-face) and anthropometric measurements (See *Extended Data*). The tablets run an application built in Open Data Kit (ODK) for data collection, a free software at the moment of the survey ^24^.

Once the questionnaires were completed, weight measurements were performed using a bioelectrical impedance device (TBF-300A, TANITA Corporation, Tokyo, Japan), and standing height using a stadiometer and standardized procedures. Blood pressure was assessed in triplicate using an OMRON HEM-780 automatized blood pressure monitor (OMRON Healthcare, Illinois, USA), previously validated for adult population ^25^.

Participants were explained about procedures for blood sample collection by trained laboratory staff. A venous blood sample (7.5 ml) was drawn from participants after a period of fasting of 8 to 12 hours. Then, a load of 75 g of anhydrous glucose in a volume of 300 ml of water was given. A new blood sample was obtained 2 hours later in order to measure postprandial glucose ^21^. Questionnaires and clinical measurements were conducted between blood samplings. Blood analyses were performed in a certified laboratory in Lima. A Cobas Modular Platform automated analyser and reagents (number of reagents used 3350, including fasting and postprandial assessments), supplied by Roche Diagnostics (catalogue number: 04404483190). Bio-Rad provided a reference range with respect to quality control for glucose measurements, where <1 was taken for the coefficient of variation.

### Statistical analysis

The software used for statistical analysis was STATA 16 for Windows (StataCorp, College Station, TX, US), and a p-value <0.05 was considered statistically significant. To describe the numerical variables, means and standard deviation (SD) were used, whereas categorical variables were reported using frequencies and proportions. To estimate cardiovascular risk (HEARTS), the *whocvdrisk* command was used, which allows calculation of the 10-year risk in 21 regions of the world ^17^. Comparison between numerical variables was done using analysis of variance (ANOVA), whereas comparison between categorical variables was conducted using Chi-squared test.

To evaluate the association of interest, mixed linear models were built to assess the changes of cardiovascular risk by glucose metabolism disorder. The hierarchical structure of the model has two different levels: the first level was defined based on the individual's repeated measures (i.e., the HEARTS CV risk score and the mean arterial pressure), whereas the second level was the study individual, considered as the only random effect in the model. Elapsed time (in years) was included as a variable that interacted with the population group to determine the change in the outcomes of interest (CV risk score and MAP) at each assessment (at baseline and follow-up). A complete-case approach was used, and the multivariable model was adjusted by age, sex, education level, socioeconomic level, health insurance, smoking, alcohol consumption, physical activity level, and fruit and vegetable intake. The results of the models were reported using coefficients (β, implying the change mean over time) and 95% confidence intervals (95% CI).

## Results

### Characteristics of the study population

**Figure 1.**
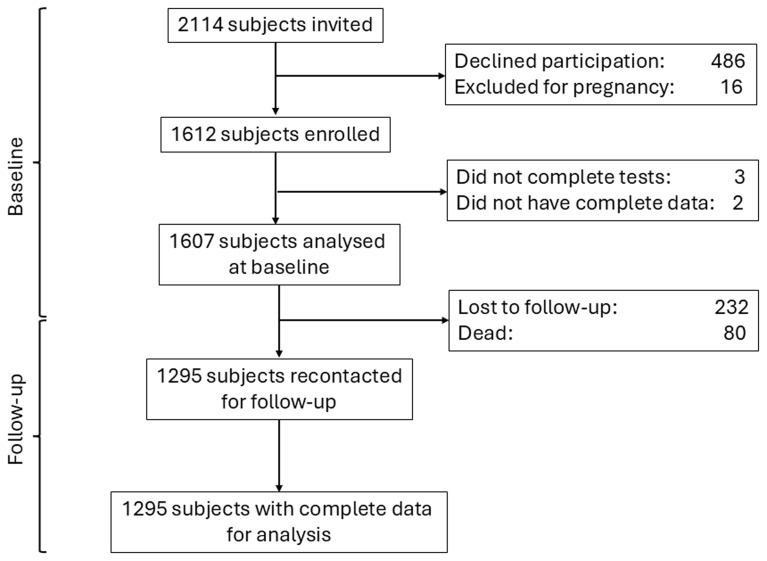
Flowchart detailing subjects’ participation at baseline and follow-up.

A total of 2114 individuals were invited to participate in the study; of them, 486 (22.9%) declined participation and 16 (0.8%) women were pregnant and were therefore excluded. Of the 1612 (76.3%) participants enrolled in the study, three did not complete the blood procedures and two did not have complete data, therefore, only 1607 subjects were analysed ( Figure 1). Of these participants, 50.3% were female, and the mean age was 48 years (SD: 10.5); while 32.2% and 46.6% had primary and secondary education, respectively.

### Characteristics of the study population at baseline according to glycaemic status

The prevalence of prediabetes at baseline was 16.9% (95% CI: 15.1% - 18.9%), while that of type 2 diabetes was 11.0% (95% CI: 9.5% - 12.6%). Sex, age, educational level, alcohol use, physical activity level, BMI and the presence of hypertension were factors associated with glycaemic status ( Table 1). Of interest, smoking and fruit or vegetable intake were not associated with the exposure of interest.

**Table 1.** Characteristics of the study population according to glucose metabolism disorder.

	Normal	Prediabetes	Type 2 diabetes	p-valor
	(n = 1159)	(n = 272)	(n = 176)	
***Sex***				<0,001
Men	612 (52.8%)	112 (41.2%)	74 (42.0%)	
Women	547 (47.2%)	160 (58.8%)	102 (58.0%)	
***Age***				<0.001
<40 years	372 (32.1%)	54 (19.9%)	14 (7.9%)	
40-49 years	354 (30.5%)	81 (29.8%)	45 (25.6%)	
50-59 years	264 (22.8%)	76 (27.9%)	69 (39.2%)	
60+ years	169 (14.6%)	61 (22.4%)	48 (27.3%)	
***Education level***				<0.001
Primary	336 (29.0%)	102 (37.5%)	80 (45.5%)	
Secondary	559 (48.2%)	116 (42.7%)	73 (41.5%)	
Superior	264 (22.8%)	54 (19.8%)	23 (13.0%)	
***Socioeconomic level***				0.29
Low	370 (31.9%)	100 (36.8%)	68 (38.6%)	
Middle	408 (35.2%)	89 (32.7%)	53 (30.1%)	
High	381 (32.9%)	83 (30.5%)	55 (31.3%)	
***Health insurance***				0.26
No	100 (9.4%)	20 (7.3%)	11 (6.2%)	
Yes	1050 (90.6%)	252 (92.7%)	165 (93.8%)	
***Smoking***				0.40
No	1087 (93.8%)	260 (95.6%)	168 (95.5%)	
Yes	72 (6.2%)	12 (4.4%)	8 (4.5%)	
***Alcohol consumption***				0.002
≤1 per month	1030 (88.9%)	259 (95.2%)	165 (93.8%)	
>1 per month	129 (11.1%)	13 (4.8%)	11 (6.2%)	
***Physical activity level***				0.02
Moderate/high	744 (64.2%)	165 (60.7%)	94 (53.4%)	
Low	415 (35.8%)	107 (39.3%)	82 (46.6%)	
***Fruits and vegetables intake***				0.85
<1 per day	548 (47.3%)	133 (48.9%)	86 (48.9%)	
≥=1 per day	611 (52.7%)	139 (51.1%)	90 (51.1%)	
***Obesity***				<0.001
No	855 (73.8%)	158 (58.1%)	118 (67.1%)	
Yes	304 (26.2%)	114 (41.9%)	58 (32.9%)	
***Hypertension***				<0.001
No	907 (78.3%)	179 (65.8%)	104 (59.1%)	
Yes	252 (21.7%)	93 (34.2%)	72 (40.9%)	

### Cardiovascular risk according to the characteristics of the study population

The mean cardiovascular risk was 3.0% (SD: 2.5%), whereas the mean arterial pressure was 93.1 (SD: 11.9) mmHg. Cardiovascular risk was higher in males, those of older age, those with lower education, with low socioeconomic status, who reported having smoked, those with higher alcohol consumption, with moderate/high physical activity, with hypertension, and with glycaemic status ( Table 2).

**Table 2.** Cardiovascular risk (HEARTS) and mean arterial pressure by characteristics of the study population.

	N	Cardiovascular risk (HEARTS, %)		Mean blood pressure (mm Hg)	
		Mean (SD)	p-value	Mean (SD)	p-value
***Sex***			<0.001		<0.001
Men	798	3.8 (2.8)		95.7 (11.2)	
Women	809	2.3 (2.0)		90.5 (12.0)	
***Age***			<0.001		<0.001
<40 years	440	1.1 (0.8)		88.5 (9.3)	
40-49 years	480	1.9 (1.2)		92.4 (11.2)	
50-59 years	409	3.7 (1.7)		95.6 (12.0)	
60+ years	278	6.8 (2.5)		97.8 (13.5)	
***Education level***			<0.001		<0.001
Primary	518	4.3 (2.8)		95.1 (12.5)	
Secondary	748	2.6 (2.3)		92.8 (11.5)	
Superior	341	1.9 (1.6)		90.6 (11.1)	
***Socioeconomic level***			0.01		0.41
Low	538	3.3 (2.6)		93.3 (11.7)	
Middle	550	2.9 (2.5)		92.5 (11.9)	
High	519	2.9 (2.5)		93.4 (12.0)	
***Health insurance***			0.59		0.29
No	140	2.9 (2.6)		92.1 (11.8)	
Yes	1467	3.0 (2.5)		93.2 (11.9)	
***Smoking***			<0.001		0.85
No	1515	2.9 (2.5)		93.1 (12.0)	
Yes	92	5.1 (2.8)		92.8 (9.5)	
***Alcohol consumption***			0.03		0.08
≤1 per month	1454	3.0 (2.5)		92.9 (11.9)	
>1 per month	153	3.4 (2.9)		94.7 (11.3)	
***Physical activity level***			0.02		0.91
Moderate/high	1003	3.1 (2.6)		93.0 (11.7)	
Low	604	2.8 (2.4)		93.1 (12.1)	
***Fruits and vegetables intake***			0.13		0.27
<1 per day	767	3.1 (2.6)		93.4 (11.8)	
≥1 per day	840	2.9 (2.4)		92.8 (11.9)	
***Obesity***			0.33		0.002
No	1131	3.0 (2.5)		92.5 (12.0)	
Yes	476	3.1 (2.5)		94.5 (11.4)	
***Hypertension***			<0.001		<0.001
No	1190	2.4 (1.9)		88.7 (7.7)	
Yes	417	4.8 (3.2)		105.6 (12.6)	
***Glycaemic status***			<0.001		<0.001
Normoglycaemia	1159	2.7 (2.4)		91.8 (11.1)	
Prediabetes	272	3.5 (2.7)		95.5 (11.9)	
Type 2 diabetes	176	4.0 (2.7)		97.6 (14.6)	

Similarly, mean arterial pressure was, on average, higher among males, those of older age, with low educational level, with obesity, with hypertension, and glycaemia status ( Table 2).

### Association between glycaemic status and cardiovascular risk

In the Table 3, our multivariable model using information from study baseline, showed that those with prediabetes and type 2 diabetes had a higher cardiovascular risk and mean blood pressure than participants with normoglycaemia.

**Table 3.** Association between glucose metabolism disorder, cardiovascular risk and mean arterial pressure at baseline: crude and adjusted models.

	Crude model	Adjusted model *
	β (95% CI)	β (95% CI)
	**Cardiovascular risk (%)**	
***Glucose metabolism disorder***		
Normoglycemia	Reference	Reference
Prediabetes	**0.71 (0.35 a 1.06)**	**0.31 (0.12 a 0.50)**
Type 2 diabetes	**1.27 (0.85 a 1.70)**	**0.33 (0.06 a 0.60)**
	**Mean arterial pressure (mmHg)**	
***Glucose metabolism disorder***		
Normoglycemia	Reference	Reference
Prediabetes	**3.63 (2.08 a 5.18)**	**3.29 (1.82 a 4.87)**
Type 2 diabetes	**5.74 (3.50 a 7.99)**	**4.22 (2.03 a 6.40)**

Values in bold are statistically significant (p<0.05) * Model adjusted for sex, age, education level, socioeconomic level, health insurance, smoking, alcohol consumption, physical activity level, and vegetable and fruit intake.

After 5.9 (SD: 0.3) years of follow-up, a total of 1295 (80.6%) participants were recontacted, had complete data and were analysed ( Figure 1). The comparison of the characteristics of those included and not included in the follow-up analysis is shown in Table 4.

**Table 4.** Comparison between those included and not included in follow-up analysis.

	Included	Not included	p-valor
	(n = 1295)	(n = 312)	
***Sex***			0.25
Men	634 (49.0%)	164 (52.6%)	
Women	661 (51.0%)	148 (47.4%)	
***Age***			<0.001
<40 years	351 (27.1%)	89 (28.5%)	
40-49 years	406 (31.3%)	74 (23.7%)	
50-59 years	339 (26.2%)	70 (22.4%)	
60+ years	199 (15.4%)	79 (25.4%)	
***Education level***			0.65
Primary	412 (31.8%)	106 (34.0%)	
Secondary	603 (46.6%)	145 (46.5%)	
Superior	280 (21.6%)	61 (19.5%)	
***Socioeconomic level***			0.19
Low	420 (32.4%)	118 (37.8%)	
Middle	451 (34.8%)	99 (31.7%)	
High	424 (32.8%)	95 (30.5%)	
***Health insurance***			0.08
No	105 (8.1%)	35 (11.2%)	
Yes	1190 (91.9%)	277 (88.8%)	
***Smoking***			0.76
No	1222 (94.4%)	293 (93.9%)	
Yes	73 (5.6%)	19 (6.1%)	
***Alcohol consumption***			0.36
≤1 per month	1176 (90.8%)	278 (89.1%)	
>1 per month	119 (9.2%)	34 (10.9%)	
***Physical activity level***			0.41
Moderate/high	802 (61.9%)	201 (64.4%)	
Low	493 (38.1%)	111 (35.6%)	
***Fruits and vegetables intake***			0.91
<1 per day	619 (47.8%)	148 (47.4%)	
≥=1 per day	676 (52.2%)	164 (52.6%)	
**Obesity**			0.74
No	909 (70.2%)	222 (71.2%)	
Yes	386 (29.8%)	90 (28.8%)	
***Hypertension***			0.67
No	956 (73.8%)	234 (75.0%)	
Yes	339 (26.2%)	78 (25.0%)	

During follow-up, crude changes in cardiovascular risk (i.e., HEARTS score and mean arterial pressure) are presented in Table 5. Overall, CV risk score increased among those with normoglycaemia but no change was found in the prediabetes and diabetes groups. On the other hand, mean arterial pressure reduced markedly in the three groups.

**Table 5.** Changes in cardiovascular risk and mean arterial pressure over time.

	Cardiovascular risk			
	At baseline		At follow-up	
*Glycaemic status*	n	Mean (SD)	n	Mean (SD)
Normoglycaemia	1159	2.7 (2.4)	933	3.4 (2.2)
Prediabetes	272	3.5 (2.7)	228	3.5 (2.1)
Type 2 diabetes	176	4.0 (2.7)	134	3.9 (2.9)
	Mean arterial pressure			
	At baseline		At follow-up	
*Glycaemic status*	N	Mean (SD)	N	Mean (SD)
Normoglycaemia	1159	91.8 (11.1)	933	81.6 (6.6)
Prediabetes	272	95.5 (11.9)	228	82.7 (7.6)
Type 2 diabetes	176	97.6 (14.6)	134	82.1 (7.7)

When the change of cardiovascular risk over time was evaluated ( Table 6), an increase in the risk was found among those with normoglycemia, but no change was seen between baseline and follow-up among those with prediabetes and type 2 diabetes. Nevertheless, there was a reduction in the mean arterial pressure, but this was greater in those with normoglycemia than in those with prediabetes and type 2 diabetes.

**Table 6.** Association between glucose metabolism disorder and change in cardiovascular risk and mean arterial pressure: crude and adjusted models.

	Crude model	Adjusted model *
	β (95% CI)	β (95% CI)
	**Change in cardiovascular risk at follow-up (%)**	
***Glycaemic status at baseline***		
Normoglycemia	**0.85 (0,76 to 0.95)**	**0.88 (0.79 to 0.98)**
Prediabetes	-0.06 (-0.31 to 0.18)	-0.06 (-0.30 to 0.16)
Type 2 diabetes	-0.16 (-0.51 to 017)	-0.18 (-0.51 to 0.13)
	**Change in mean arterial pressure at follow-up (mmHg)**	
***Glycaemic status at baseline***		
Normoglycemia	**-10.20 (-10.94 to -9.45)**	**-10.11 (-10.86 to -9.36)**
Prediabetes	**-2.61 (-4.44 to -0.78)**	**-2.62 (-4.45 to -0.78)**
Type 2 diabetes	**-5.31 (-8.09 to -2.52)**	**-5.42 (-8.21 to -2.64)**

Values in bold are statistically significant (p<0.05) * Model adjusted for sex, age, education level, socioeconomic level, health insurance, smoking, alcohol consumption, physical activity level, and vegetable and fruit intake.

## Discussion

### Main findings

Our multivariable model shows that, at the beginning of the cohort, cardiovascular risk was higher among those with type 2 diabetes than in those with prediabetes, and both groups had a higher cardiovascular risk than those with normoglycaemia. The findings were similar in the case of mean blood pressure, which was higher in people with type 2 diabetes and prediabetes than in those with normoglycaemia. At follow-up, an increase in cardiovascular risk was observed among those with normoglycaemia and no change in those with prediabetes or type 2 diabetes; however, mean arterial pressure had a marked reduction in the three groups, which was more patent among those with normoglycemia at baseline.

### Comparison with previous studies

The high cardiovascular risk associated with type 2 diabetes has already been described in previous studies. For example, a systematic review including 57 articles reported that cardiovascular disease affects 32% of people living with diabetes, being one of the major causes of mortality ^26^. In the case of prediabetes, a systematic review including 53 prospective studies and data from 1.6 million individuals reported that prediabetes, assessed using fasting glucose, postprandial glucose or glycosylated haemoglobin, was associated with increased cardiovascular risk ^27^. Thus, the results of our study, at least at the baseline of the cohort, are in relation to previous literature.

Under the same line, a population-based cohort study involving more than 100,000 participants from 20 diverse communities in China concluded that participants with prediabetes and diabetes with 5 or more ideal markers of cardiovascular health had lower excess cardiovascular risk compared with participants with normal glycaemia ^13^. Thus, these findings highlight the need to have glycaemia and cardiovascular health markers (e.g., blood pressure or cholesterol) controlled in individuals with prediabetes, and more importantly among those with type 2 diabetes diagnosis.

Regarding blood pressure, a cohort study conducted in Japan sought to determine the association of SBP and DBP with the occurrence of a cardiovascular event according to glycaemia status ^28^. The results indicated that cardiovascular risk increased gradually with increasing SBP and DBP regardless of the degree of glycaemia abnormality. Another study, using data from the US National Health and Nutrition Examination Survey (NHANES) reported a U-shaped relationship between SBP and all-cause mortality regardless of diabetes diagnosis, highlighting the need for appropriate control of blood pressure levels to reduce cardiovascular risk. A study conducted in South Korea analysed data from 5413 participants and reported that progression from a specific glycaemic status to another (i.e., progression from normal glycemia to prediabetes, or from prediabetes to diabetes) was a determinant of increased blood pressure and the development of hypertension ^12^.

Locally, a prospective cohort study conducted in Peru, analysing data from 3237 participants showed an increase of 2.8 mmHg in SBP and 1.5 mmHg in DBP in subjects with type 2 diabetes after 2.5 years of follow-up ^29^. Whilst our work evidenced a reduction in mean arterial pressure levels in all three study groups, the reduction was smaller in those with prediabetes and type 2 diabetes. Such drop in blood pressure levels found in our work could be due to several factors, including regression to the mean (i.e., if one sample of a random variable is extreme, the next sampling of the same random variable is likely to be closer to its mean ^30^); reduction in the number of cases with prediabetes and diabetes, as well as those with risk factors such as obesity, due to the COVID-19 pandemic that occurred between the two evaluations; or appropriate control of blood pressure and glycaemia during the same pandemic period. In addition, glucose status transition, i.e., from prediabetes to overt diabetes or from prediabetes to normoglycaemia, may influence our outcomes. These changes over time were not assessed as the follow-up did not include glucose evaluation. In any case, the mean arterial pressure level in the study was always higher among those with glucose metabolism disorder than among those without it.

### Relevance of findings

The study was conducted in Tumbes, a region of a developing country, where few studies have been carried out to associate glucose metabolism disorders (prediabetes and type 2 diabetes) and cardiovascular risk. Our findings show that those with prediabetes and type 2 diabetes have higher mean arterial pressure than those with normoglycaemia, and this finding is maintained even six years later. This should be considered for appropriate management of these patients.

Even though prediabetes is highly prevalent, there are usually no procedures for appropriate detection or management in Peru. Even national guidelines focus on the detection, treatment and management of patients with type 2 diabetes ^14^, but not on those with prediabetes. It is possible that, as patients with type 2 diabetes, those with prediabetes require multidisciplinary management, including cardiovascular, neurologic, ophthalmologic, renal and other evaluations.

In addition, estimating a subject’s cardiovascular risk may require a series of laboratory tests (fasting glucose and total cholesterol), and these are not always available in primary care settings. Our findings show that blood pressure measurement and a laboratory-free cardiovascular risk score could help in the detection and perhaps appropriate management of these patients.

### Strengths and limitations

This is a prospective study that allows us to establish temporality between the variables of interest over time. In addition, we used the HEARTS (without laboratory tests), which is a WHO-validated score adaptable to different contexts. However, our study has limitations that deserve discussion. First, as an observational study, only association and not causality can be determined. Second, a potential selection bias may be present since the area chosen for the study does not represent all of Peru and was selected because high prevalence of obesity and type 2 diabetes. Memory and social desirability bias could be present, especially in the case of variables such as tobacco, used for estimation of cardiovascular risk, and alcohol consumption. Third, prediabetes is a state that can progress towards type 2 diabetes or revert to normoglycaemia ^31^; so that in the 6-year follow-up, the change in the condition could affect the final cardiovascular risk. It was not possible to measure glycaemic control at baseline or glycaemic status at follow-up. Fourth, potential confounders (i.e. diet patterns, salt intake, etc.) and intercurrent factors (i.e. anti-hypertensive, glucose-lowering and lipid-lowering medication) were not assessed and for instance, not included in the analysis, and may plausibly influence both mean arterial pressure and cardiovascular risk. Finally, many participants were lost to follow-up and could not be contacted because they migrated from the study area due to economic crisis and mortality because of the COVID-19 pandemic. Nevertheless, the only difference between those included and those not included in the follow-up analysis was age, so those older were lost to follow-up.

## Conclusions

This study identified that glucose metabolism disorders are associated with increased cardiovascular risk and mean arterial blood pressure at baseline, and high mean arteria blood pressure after six years. These findings suggest the need for more comprehensive cardiovascular risk monitoring in patients with prediabetes and type 2 diabetes.

## Ethics and consent

The present study adhered to the Declaration of Helsinki. The protocol, informed consent and tools used at the study baseline were reviewed and approved by the Institutional Ethics Committees (EIC) of the Universidad Peruana Cayetano Heredia, in Lima, Peru (SIDISI code: 63585, date of approval: February 10, 2015), and the London School of Hygiene and Tropical Medicine (LSHTM), in London, UK (code: 11783, date of approval: October 3, 2016). A written informed consent was read before enrolment to ensure participation.

The protocol, informed consent and tools used in the follow-up were approved by the EIC of the Universidad Peruana Cayetano Heredia (SIDISI code: 209685, date of approval: November 2, 2022). Similar to the baseline, a written informed consent was used before ensure participation.

The present analysis was reviewed and exempted from review by the Research Ethics Committee of the Universidad Científica del Sur.

## Data Availability

Figshare: T2DM and anxiety, https://doi.org/10.6084/m9.figshare.16862191.v2 ^32^ This project contains the following underlying data: - T2DM and anxiety v11.csv (dataset) - Dictionary (110521).txt (key to variable abbreviations) Data are available under the terms of the Creative Commons Attribution 4.0 International license (CC-BY 4.0). Figshare: T2DM SCREEN – Questionnaires (baseline and follow-up), https://doi.org/10.6084/m9.figshare.26348926.v1 ^33^ Data are available under the terms of the Creative Commons Attribution 4.0 International license (CC-BY 4.0).

## Software availability

-    OpenEpi, a free and open-source software for epidemiologic statistics, was used for sample size calculation. Available at: www.openepi.com

-    HEARTS en las Américas: Estimar el riesgo cardiovascular (calculadora de riesgo). Available at: https://www.paho.org/cardioapp/web/#/cvrisk

-    Open Data Kit (ODK) for data collection, a free software at the moment of the survey, currently it is not. Available at: https://getodk.org/
