# Supplementary material for: Glucose metabolism disorders and changes in cardiovascular risk among adult Peruvian population
Source: Wellcome Open Res. 2026 Mar 9;9:442. Originally published 2024 Aug 12. [Version 3] doi: 10.12688/wellcomeopenres.22701.3 (PMC13040229; doi:10.12688/wellcomeopenres.22701.3)
Supplement: Peer review history

# Reviewer report: Reviewer response for version 3

**DOI:** 10.21956/wellcomeopenres.28640.r150309

**Published:** 2026-03-31

**Authors:** Ray Ticse

**Affiliations:** 1 Universidad Peruana Cayetano Heredia Facultad de Medicina, Lima District, Lima Region, Peru

The concerns I initially raised have been adequately addressed by the authors. In my opinion, the manuscript can proceed with the editorial process and indexing

Is the work clearly and accurately presented and does it cite the current literature?

Yes

If applicable, is the statistical analysis and its interpretation appropriate?

Yes

Are all the source data underlying the results available to ensure full reproducibility?

Yes

Is the study design appropriate and is the work technically sound?

Yes

Are the conclusions drawn adequately supported by the results?

Yes

Are sufficient details of methods and analysis provided to allow replication by others?

Yes

Reviewer Expertise:

Endocrine disorders

I confirm that I have read this submission and believe that I have an appropriate level of expertise to confirm that it is of an acceptable scientific standard.

# Reviewer report: Reviewer response for version 3

**DOI:** 10.21956/wellcomeopenres.28640.r150308

**Published:** 2026-03-10

**Authors:** Paul Anthony Camacho Lopez

**Affiliations:** 1 FOSCAL Internacional, Bucaramanga, Colombia

Approved

Is the work clearly and accurately presented and does it cite the current literature?

Yes

If applicable, is the statistical analysis and its interpretation appropriate?

Partly

Are all the source data underlying the results available to ensure full reproducibility?

Partly

Is the study design appropriate and is the work technically sound?

Yes

Are the conclusions drawn adequately supported by the results?

Partly

Are sufficient details of methods and analysis provided to allow replication by others?

Partly

Reviewer Expertise:

Cardiovascular epidemiology

I confirm that I have read this submission and believe that I have an appropriate level of expertise to confirm that it is of an acceptable scientific standard.

# Reviewer report: Reviewer response for version 2

**DOI:** 10.21956/wellcomeopenres.27863.r140946

**Published:** 2026-01-31

**Authors:** Ray Ticse

**Affiliations:** 1 Universidad Peruana Cayetano Heredia Facultad de Medicina, Lima District, Lima Region, Peru

Major observations

1. **Objective and longitudinal interpretation**
  The study evaluates changes in cardiovascular risk over time according to glucose metabolism status; however, glycaemic status was assessed only at baseline. Given the dynamic nature of prediabetes, this limits the interpretation of longitudinal findings. The objective and conclusions should more clearly state that analyses are based on baseline glycaemic status rather than on glycaemic progression.
2. **Interpretation of longitudinal findings**
  The increase in cardiovascular risk observed only among normoglycaemic participants, together with the reduction in mean arterial pressure across all groups, is unexpected. These findings should be interpreted more cautiously and framed as descriptive, considering potential explanations such as regression to the mean, unmeasured treatments, behavioural changes, or the impact of the COVID-19 period.
3. **Unmeasured intercurrent factors**
  The absence of information on antihypertensive, glucose-lowering and lipid-lowering therapies, as well as on changes in weight, diet or glycaemic control during follow-up, represents an important limitation. These factors could substantially influence both cardiovascular risk estimates and blood pressure outcomes and should be more explicitly integrated into the discussion.
4. **Loss to follow-up**
  Approximately 20% of participants were lost to follow-up, and those lost were older. This raises concerns about potential attrition or survivor bias and its impact on longitudinal estimates, which should be discussed more explicitly.

Minor observations

1. **Outcome overlap**
  Systolic blood pressure is included in the WHO HEARTS score, while mean arterial pressure is analysed separately. This partial overlap should be acknowledged to avoid misinterpretation.
2. **Presentation and clarity**
  Clarify in tables whether longitudinal analyses include only participants with complete paired data. A simple figure showing trends in cardiovascular risk and mean arterial pressure over time could improve readability.
3. **Language consistency**
  Ensure consistent terminology (e.g., “normoglycaemia” vs. “normoglycemia”) and correct minor typographical errors.

Is the work clearly and accurately presented and does it cite the current literature?

Yes

If applicable, is the statistical analysis and its interpretation appropriate?

Yes

Are all the source data underlying the results available to ensure full reproducibility?

Yes

Is the study design appropriate and is the work technically sound?

Yes

Are the conclusions drawn adequately supported by the results?

Yes

Are sufficient details of methods and analysis provided to allow replication by others?

Yes

Reviewer Expertise:

Endocrine disorders

I confirm that I have read this submission and believe that I have an appropriate level of expertise to confirm that it is of an acceptable scientific standard, however I have significant reservations, as outlined above.

# Response

**Published:** 2026-02-03

**Authors:** Antonio Bernabe-Ortiz

**Affiliations:** Universidad Cientifica del Sur, Peru

**Major observations**

**1. Objective and longitudinal interpretation** The study evaluates changes in cardiovascular risk over time according to glucose metabolism status; however, glycaemic status was assessed only at baseline. Given the dynamic nature of prediabetes, this limits the interpretation of longitudinal findings. The objective and conclusions should more clearly state that analyses are based on baseline glycaemic status rather than on glycaemic progression. **Response:** We have rephrased several sections in the introduction, methods, and discussion (conclusion) to clearly state that results are based on glycaemic status at baseline.  

**2. Interpretation of longitudinal findings** The increase in cardiovascular risk observed only among normoglycaemic participants, together with the reduction in mean arterial pressure across all groups, is unexpected. These findings should be interpreted more cautiously and framed as descriptive, considering potential explanations such as regression to the mean, unmeasured treatments, behavioural changes, or the impact of the COVID-19 period. **Response:** We have expanded our Discussion section to include potential explanations as requested by the reviewer. Now, it reads: “ *Thus, such drop in blood pressure levels found in our work could be due to several factors, including regression to the mean (i.e., if one sample of a random variable is extreme, the next sampling of the same random variable is likely to be closer to its mean ^30^ ); reduction in the number of cases with prediabetes and diabetes, as well as those with risk factors such as obesity, due to the COVID-19 pandemic that occurred between the two evaluations; unmeasured treatments (those with treatment for hypertension, diabetes or dyslipidaemia) or appropriate control of blood pressure and glycaemia during the same pandemic period*”.  

**3. Unmeasured intercurrent factors** The absence of information on antihypertensive, glucose-lowering and lipid-lowering therapies, as well as on changes in weight, diet or glycaemic control during follow-up, represents an important limitation. These factors could substantially influence both cardiovascular risk estimates and blood pressure outcomes and should be more explicitly integrated into the discussion.

**Response:** We have expanded the limitation section to include this: “ *Fourth, potential confounders (i.e. diet patterns, salt intake, etc.) and unmeasured intercurrent factors (i.e. anti-hypertensive, glucose-lowering and lipid-lowering medication as well as changes in weight, diet or glycaemic control during follow-up) were not assessed and for instance, not included in the analysis, and may plausibly influence both mean arterial pressure and cardiovascular risk*”.  

**4. Loss to follow-up** Approximately 20% of participants were lost to follow-up, and those lost were older. This raises concerns about potential attrition or survivor bias and its impact on longitudinal estimates, which should be discussed more explicitly.

**Response:** We have expanded this section to include the points suggested by the reviewer: “ *Finally, many participants were lost to follow-up arising potential attrition bias. Moreover, some study subjects were not contacted because they migrated from the study area due to economic crisis and mortality associated with the COVID-19 pandemic. As those older were more probable to be lost to follow-up, survivor bias may be an issue*”.  

**Minor observations**  

**1. Outcome overlap** Systolic blood pressure is included in the WHO HEARTS score, while mean arterial pressure is analysed separately. This partial overlap should be acknowledged to avoid misinterpretation.

**Response:** That is correct. We are using two different markers of cardiovascular risk, and both are based on blood pressure. We have acknowledged that in our Methods (Definition of variables) section: “ *Two were our study outcomes related to cardiovascular risk, and both included blood pressure indicators (i.e., a cardiovascular risk score and mean arterial pressure)*”.  

**2. Presentation and clarity** Clarify in tables whether longitudinal analyses include only participants with complete paired data. A simple figure showing trends in cardiovascular risk and mean arterial pressure over time could improve readability.

**Response:** We have included that information in the statistical analysis section: “ *A complete-case approach was used, and the multivariable model was adjusted by age, sex, education level, socioeconomic level, health insurance, smoking, alcohol consumption, physical activity level, and fruit and vegetable intake*”, as well as in tables 3 and 6 as requested: “ *A complete-case approach was used, and model was adjusted for sex, age, education level, socioeconomic level, health insurance, smoking, alcohol consumption, physical activity level, and vegetable and fruit intake*”.  

**3. Language consistency** Ensure consistent terminology (e.g., “normoglycaemia” vs. “normoglycemia”) and correct minor typographical errors.

**Response:** We have reviewed the whole manuscript to avoid inconsistencies and correct mistakes.

# Reviewer report: Reviewer response for version 2

**DOI:** 10.21956/wellcomeopenres.27863.r139337

**Published:** 2025-11-22

**Authors:** Paul Anthony Camacho Lopez

**Affiliations:** 1 FOSCAL Internacional, Bucaramanga, Colombia

I agree with the revisions. I believe it's a relevant article for cardiovascular health.

Is the work clearly and accurately presented and does it cite the current literature?

Yes

If applicable, is the statistical analysis and its interpretation appropriate?

Partly

Are all the source data underlying the results available to ensure full reproducibility?

Partly

Is the study design appropriate and is the work technically sound?

Yes

Are the conclusions drawn adequately supported by the results?

Partly

Are sufficient details of methods and analysis provided to allow replication by others?

Partly

Reviewer Expertise:

Cardiovascular epidemiology

I confirm that I have read this submission and believe that I have an appropriate level of expertise to confirm that it is of an acceptable scientific standard.

# Reviewer report: Reviewer response for version 1

**DOI:** 10.21956/wellcomeopenres.25003.r135359

**Published:** 2025-10-31

**Authors:** Paul Anthony Camacho Lopez

**Affiliations:** 1 FOSCAL Internacional, Bucaramanga, Colombia

This longitudinal cohort study, conducted in Tumbes, Peru, tracked adults over roughly six years to explore whether initial glycemic status might predict later shifts in cardiovascular risk—assessed using the non-laboratory-based WHO HEARTS score—as well as changes in mean arterial pressure (MAP). The findings suggest that individuals with prediabetes or diabetes started off with both higher cardiovascular risk and elevated MAP. Over time, however, absolute risk changes were modest, and MAP declined across all groups. Given the growing importance of chronic disease management in resource-constrained settings, the research question is highly relevant. Moreover, the study’s prospective design and use of standardized tools add methodological strength.

 That said, the application of mixed-effects models needs further elaboration. Details on the structure—such as the specification of random effects, how time was coded, whether an interaction between time and glycemic status was incorporated, and the choice of covariance structure—are essential for interpretability and reproducibility. Without these elements, it's difficult to assess the robustness of the findings.

 The issue of loss to follow-up also deserves greater transparency. A participant flowchart, along with a comparative table showing baseline characteristics for those reassessed versus those lost, would be valuable for evaluating potential selection bias. Concerning missing data, the justification for using a complete-case approach should be made explicit. If possible, a sensitivity analysis could strengthen the credibility of the conclusions.

 Intercurrent factors—such as antihypertensive, glucose-lowering, and lipid-lowering therapies, along with behavioral changes—could plausibly influence both MAP and cardiovascular risk. These need to be described more thoroughly, as their presence or absence could substantially affect interpretation.

 Lastly, the lack of follow-up data on glycemic status warrants clearer acknowledgment. Participants likely experienced status transitions (e.g., from prediabetes to overt diabetes), and such shifts may have influenced outcomes. Discussing these transitions explicitly would contextualize the findings. Including a table that summarizes baseline and follow-up HEARTS and MAP values by glycemic group, along with a trajectory plot, would also enhance clarity regarding the clinical relevance of the observed changes.

Is the work clearly and accurately presented and does it cite the current literature?

Yes

If applicable, is the statistical analysis and its interpretation appropriate?

Partly

Are all the source data underlying the results available to ensure full reproducibility?

Partly

Is the study design appropriate and is the work technically sound?

Yes

Are the conclusions drawn adequately supported by the results?

Partly

Are sufficient details of methods and analysis provided to allow replication by others?

Partly

Reviewer Expertise:

Cardiovascular epidemiology

I confirm that I have read this submission and believe that I have an appropriate level of expertise to confirm that it is of an acceptable scientific standard, however I have significant reservations, as outlined above.

# Response

**Published:** 2025-11-04

**Authors:** Antonio Bernabe-Ortiz

**Affiliations:** Universidad Cientifica del Sur, Peru

**This longitudinal cohort study, conducted in Tumbes, Peru, tracked adults over roughly six years to explore whether initial glycemic status might predict later shifts in cardiovascular risk—assessed using the non-laboratory-based WHO HEARTS score—as well as changes in mean arterial pressure (MAP). The findings suggest that individuals with prediabetes or diabetes started off with both higher cardiovascular risk and elevated MAP. Over time, however, absolute risk changes were modest, and MAP declined across all groups. Given the growing importance of chronic disease management in resource-constrained settings, the research question is highly relevant. Moreover, the study’s prospective design and use of standardized tools add methodological strength.** **Response:** Thanks to the reviewer for the comments. **That said, the application of mixed-effects models needs further elaboration. Details on the structure—such as the specification of random effects, how time was coded, whether an interaction between time and glycemic status was incorporated, and the choice of covariance structure—are essential for interpretability and reproducibility. Without these elements, it's difficult to assess the robustness of the findings.**

**Response:** We have expanded this section to clarify reviewer’s comments. Now it reads: “ *To evaluate the association of interest, mixed linear models were built to assess the changes of cardiovascular risk by glucose metabolism disorder. The hierarchical structure of the model has two different levels: the first level was defined based on the individual's repeated measures (i.e., the HEARTS CV risk score and the mean arterial pressure), whereas the second level was the study individual, considered as the only random effect in the model. Elapsed time (in years) was included as a variable that interacted with the population group to determine the change in the outcomes of interest (CV risk score and MAP) at each assessment (at baseline and follow-up). A complete-case approach was used, and the multivariable model was adjusted by age, sex, education level, socioeconomic level, health insurance, smoking, alcohol consumption, physical activity level, and fruit and vegetable intake. The results of the models were reported using coefficients (β, implying the change mean over time) and 95% confidence intervals (95% CI)*”.

**The issue of loss to follow-up also deserves greater transparency. A participant flowchart, along with a comparative table showing baseline characteristics for those reassessed versus those lost, would be valuable for evaluating potential selection bias. Concerning missing data, the justification for using a complete-case approach should be made explicit. If possible, a sensitivity analysis could strengthen the credibility of the conclusions.**

**Response:** For sake of transparency, we are including a flow-chart with details as requested. In addition, we have added a table (Table 4) comparing baseline characteristics between those included and those not included in the follow-up analysis. For follow-up analysis a complete-case approach was used, and the information was now added in the Statistical analysis section. We considered the possibility of conducting a multiple imputation approach; however, the outcome may not be missing completely at random or at random.

**Intercurrent factors—such as antihypertensive, glucose-lowering, and lipid-lowering therapies, along with behavioral changes—could plausibly influence both MAP and cardiovascular risk. These need to be described more thoroughly, as their presence or absence could substantially affect interpretation.**

**Response:** This topic has been included as part of the limitations section. Mow it reads: “ *Fourth, potential confounders (i.e. diet patterns, salt intake, etc.) and intercurrent factors (i.e. anti-hypertensive, glucose-lowering and lipid-lowering medication) were not assessed and for instance, not included in the analysis, and may plausibly influence both mean arterial pressure and cardiovascular risk*”.

**Lastly, the lack of follow-up data on glycemic status warrants clearer acknowledgment. Participants likely experienced status transitions (e.g., from prediabetes to overt diabetes), and such shifts may have influenced outcomes. Discussing these transitions explicitly would contextualize the findings. Including a table that summarizes baseline and follow-up HEARTS and MAP values by glycemic group, along with a trajectory plot, would also enhance clarity regarding the clinical relevance of the observed changes.** **Response:** We have included extra information regarding glucose status transition. Now it reads: “ *In addition, glucose status transition, i.e., from prediabetes to overt diabetes or from prediabetes to normoglycaemia, may influence our outcomes. These changes over time were not assessed as the follow-up did not include glucose evaluation.*” A table summarizing baseline and follow-up estimates of cardiovascular risk is now included in the manuscript as requested by the reviewer (see below).  

**Table 5.** Changes in cardiovascular risk and mean arterial pressure over time. **Cardiovascular risk** **At baseline** **At follow-up** ***Glycaemic status*** **n** **Mean (SD)** **n** **Mean (SD)** Normoglycaemia 1159 2.7 (2.4) 933 3.4 (2.2) Prediabetes 272 3.5 (2.7) 228 3.5 (2.1) Type 2 diabetes 176 4.0 (2.7) 134 3.9 (2.9)  

**Mean arterial pressure** **At baseline** **At follow-up** ***Glycaemic status*** **N** **Mean (SD)** **N** **Mean (SD)**   Normoglycaemia 1159 91.8 (11.1) 933 81.6 (6.6)   Prediabetes 272 95.5 (11.9) 228 82.7 (7.6)   Type 2 diabetes 176 97.6 (14.6) 134 82.1 (7.7)
